# Interaction of Cationic Carbosilane Dendrimers and Their siRNA Complexes with MCF-7 Cells

**DOI:** 10.3390/ijms22137097

**Published:** 2021-07-01

**Authors:** Kamila Białkowska, Katarzyna Miłowska, Sylwia Michlewska, Paulina Sokołowska, Piotr Komorowski, Tania Lozano-Cruz, Rafael Gomez-Ramirez, Francisco Javier de la Mata, Maria Bryszewska

**Affiliations:** 1 Department of General Biophysics, Faculty of Biology and Environmental Protection, University of Lodz, 141/143 Pomorska St., 90-236 Lodz, Poland; katarzyna.milowska@biol.uni.lodz.pl (K.M.); maria.bryszewska@biol.uni.lodz.pl (M.B.); 2 Molecular and Nanostructural Biophysics Laboratory, “Bionanopark” Ldt., 114/116 Dubois St., 93-465 Lodz, Poland; paulina.sokolowska@umed.lodz.pl (P.S.); p.komorowski@bionanopark.pl (P.K.); 3 Laboratory of Microscopic Imaging and Specialized Biological Techniques, Faculty of Biology and Environmental Protection, University of Lodz, Banacha12/16, 90-237 Lodz, Poland; sylwia.michlewska@biol.uni.lodz.pl; 4 Department of Pharmacology and Toxicology, Medical University of Lodz, Żeligowskiego St. 7/9, 90-752 Lodz, Poland; 5 Department of Biophysics, Institute of Materials Science, Lodz University of Technology, 1/15 Stefanowskiego St., 90-924 Lodz, Poland; 6 Department of Organic and Inorganic Chemistry, IQAR, University of Alcalá, 28805 Madrid, Spain; tania.lozano@uah.es (T.L.-C.); rafael.gomez@uah.es (R.G.-R.); javier.delamata@uah.es (F.J.d.l.M.); 7 Networking Research Center on Bioengineering, Biomaterials and Nanomedicine (CIBER-BBN), 28029 Madrid, Spain

**Keywords:** siRNA, carbosilane dendrimers, dendriplexes, nanocarriers

## Abstract

The application of siRNA in gene therapy is mainly limited because of the problems with its transport into cells. Utilization of cationic dendrimers as siRNA carriers seems to be a promising solution in overcoming these issues, due to their positive charge and ability to penetrate cell membranes. The following two types of carbosilane dendrimers were examined: CBD-1 and CBD-2. Dendrimers were complexed with pro-apoptotic siRNA (Mcl-1 and Bcl-2) and the complexes were characterized by measuring their zeta potential, circular dichroism and fluorescence of ethidium bromide associated with dendrimers. CBD-2/siRNA complexes were also examined by agarose gel electrophoresis. Both dendrimers form complexes with siRNA. Moreover, the cellular uptake and influence on the cell viability of the dendrimers and dendriplexes were evaluated using microscopic methods and XTT assay on MCF-7 cells. Microscopy showed that both dendrimers can transport siRNA into cells; however, a cytotoxicity assay showed differences in the toxicity of these dendrimers.

## 1. Introduction

One of the most common causes of death in the world is cancer. Finding new methods of treatment is a big challenge because of heterogeneity and metastasis [1,2]. Tumor development is associated with the dysfunctional regulation of apoptosis, the process responsible for the removal of damaged or infected cells. The main regulators of apoptosis are proteins belonging to the Bcl-2 family, consisting of pro- and anti-apoptotic proteins [3]. Among the proteins that can promote cell death are, e.g., Bax, Bak and Bcl-XS, whereas among the proteins promoting cell survival are Bcl-2, Bcl-XL and Mcl-1 [4]. The inhibition or promotion of apoptosis may be due to an imbalance between these proteins [3]. The transformation of normal cells into cancerous ones leads to their uncontrolled proliferation and inability to initiate apoptosis [4,5]. One possibility for reversing this process and inducing apoptosis is a cellular mechanism that enables selective pro-survival gene silencing, called RNA interference (RNAi). This mechanism promotes messenger RNA (mRNA) degradation using small interfering RNAs (siRNAs) [6,7,8]. For this reason, siRNA is under investigation for use in gene therapy [9].

An siRNA is a small double stranded molecule, with the ability to silence genes. It is incorporated in the RISC (RNA-induced silencing complex), which mediates mRNA binding and cleavage [10].

The process of RNA interference was first observed in worms. It is believed that a function of RNAi is to protect the genome against invasive genetic material, such as viruses [10]. However, there are some limitations in using naked siRNA for gene silencing [5,10,11]. One is its inability to penetrate cell membranes due to its anionic charge and repulsion from anionic membrane surfaces [10,11]. Furthermore, siRNA effectiveness in vitro and in vivo is limited because of its low enzymatic resistance. This means that effective gene transport into target cells is the main limitation in gene therapy [5,10,11]. Therefore, the application of new synthetic particles as non-viral gene carriers could be very helpful in transporting siRNAs into cells.

Among the nanomaterials considered for potential use in siRNA transport into cells, the most promising are dendrimers [12]. Dendrimers are synthetic polymers with a diameter of 3–10 nm. The name dendrimer originates from Greek “dendron”, which means a tree and is due to their branched structure. The shape of dendrimers is similar to a sphere [13,14,15]. Generally, their structure can be divided into the following three parts: (1) a central core, which defines the interior size and the number of branches; (2) repetitive branch units for the regulation of molecular size, generation and flexibility; and (3) the terminal groups, which provide the possibility for interaction with a range of compounds [16]. The characteristic structure of dendrimers allows them to complex with different particles, because of the empty spaces within the dendrimer structure [13,14,15].

Dendrimers have a lot of advantages as effective gene carriers, e.g., their stability and/or large number of groups for nucleic acid binding [5,17]. Cationic dendrimers can penetrate cell membranes due to the positive charge on their surface, in contrast to anionic or neutral dendrimers. Moreover, cationic dendrimers can bind anionic nucleic acids forming dendriplexes, and this allows genes to be transported into cells effectively [5,17,18,19].

Many types of dendrimers have been investigated for siRNA transport, including poly(amidoamine) dendrimers (PAMAM), carbosilane dendrimers (CBD), poly(propylene imine) dendrimers, poly(L-lysine) dendrimers, triazine dendrimers, po-lyglycerol-based dendrimers and nanocarbon-based dendrimers [16]. Carbosilane dendrimers complex siRNA via electrostatic interactions and protect them against RNase degradation. CBD/siRNA complexes transfected lymphocytes and silenced the expression of glyceraldehyde 3-phosphate dehydrogenase (GAPDH), and reduced HIV replication in human leukemia T lymphocytes and primary peripheral blood mononuclear cells (PBMC), with low cytotoxicity. CBD can also transfer siRNA to neurons and block HIF1-α synthesis. These effects were similar to that achieved by viral vectors. Furthermore, the gene silencing by CBD/siRNA complexes occurred even after passing the blood–brain barrier in an in vitro model [16].

The present work shows that the new second generation carbosilane dendrimers can provide a potential delivery system to transport pro-apoptotic siRNA to target (cancer) cells. The two types of dendrimers used in this study, CBD-1 and CBD-2, differ in the type of ammonium group on their surfaces; therefore, the aim has been to verify which one is the better carrier for siRNA transport. Dendrimers were complexed with the following pro-apoptotic siRNAs: Mcl-1 and Bcl-2. The formed complexes were characterized by their zeta potential, circular dichroism (CD) and fluorescence measurements. CBD-2/siRNA complexes were also examined by agarose gel electrophoresis. Additionally, the cytotoxicity of dendrimers and formed complexes on the MCF-7 cell line and the cellular uptake of dendriplexes were investigated.

## 2. Results

### 2.1. Zeta Potential

**Figure 1:**
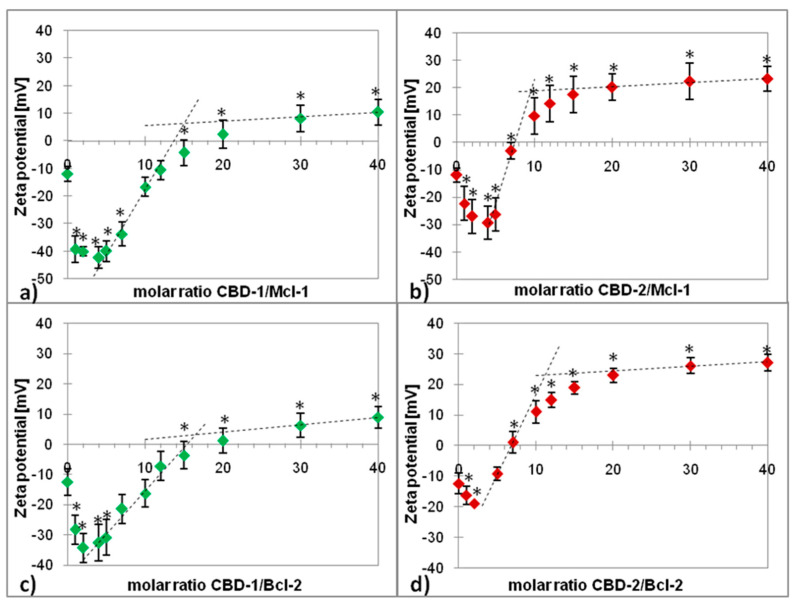
Zeta potential of the Mcl-1 (**a**,**b**) and Bcl-2 (**c**,**d**) after addition of carbosilane dendrimers CBD-1 or CBD-2 at increasing dendrimer/siRNA molar ratios in 10 mmol/L Na-phosphate buffer, pH 7.4. Results are presented as mean ± SD from a minimum of 3 independent experiments. * *p* < 0.05 compared to siRNA.

The measurement of zeta potential was used to assess changes in the surface charge of the complexes and interactions between dendrimers and siRNAs. The siRNA concentration was constant at 0.5 μM, whereas dendrimers were added in increasing concentrations. Figure 1 shows the changes in the zeta potential of siRNA after the addition of dendrimers. Both dendrimers affected the zeta potential of the selected siRNAs. The siRNAs had negative zeta potential values (both Mcl-1 and Bcl-2: ~−12 mV). The dendrimers changed the initial zeta potential values of siRNAs from negative to positive. For CBD-1, the plateau was achieved above +5 mV and for CBD-2 above +20 mV.

The two tangents method helped to determine the number of dendrimer molecules (n) that attach to one molecule of siRNA. Calculated on the basis of the graphs, n is 15 for CBD-1 and Mcl-1, 9 for CBD-2 and Mcl-1, 16 for CBD-1 and Bcl-2 and 11 for CBD-2 and Bcl-2 (Figure 1). Partial deprotonation of tertiary ammonium groups (RNHMe2) is possible at the assay pH, resulting in a lower cationic density on the surface of CBD-1. This feature could explain the higher number of molecules required to conjugate the siRNA compared to CBD-2.

### 2.2. Circular Dichroism

**Figure 2:**
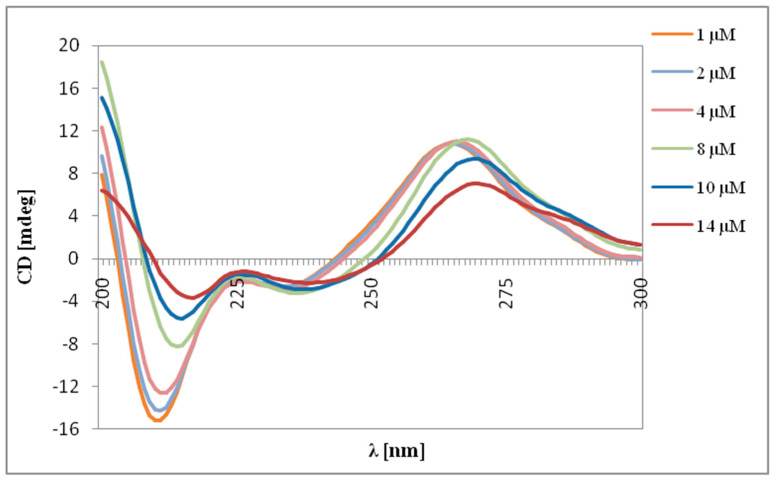
CD spectra of the Bcl-2 (2 μM) in the presence of CBD-2 dendrimer (1–14 μM) in 10 mmol/L phosphate buffer.

Changes in the secondary structure of the siRNAs (Mcl-1 and Bcl-2) under the influence of carbosilane dendrimers were also examined using CD spectroscopy. CD spectra were recorded at a wavelength range of λ = 200–300 nm. The spectra for both siRNAs were typical of the A-form, the secondary structure of RNA. They had characteristic peaks at ~210 and ~265 nm. The changes in the shape of both siRNAs’ spectra under the influence of dendrimers were also noted. The signal intensity decreased in all cases for λ = 210 nm and λ = 265 nm. We also found that dendrimers caused a shift toward longer wavelengths of both peaks. Figure 2 gives an example of changes in the spectrum for a system CBD-2/Bcl-2.

**Figure 3:**
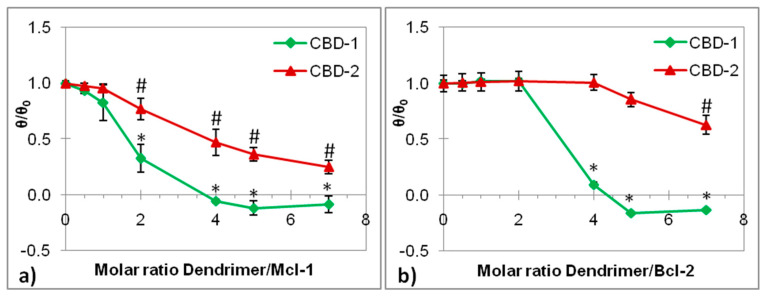
Changes in mean residue ellipticity of Mcl-1 (**a**) and Bcl-2 (**b**) at λ = 265 nm in the presence of the following dendrimers: CBD-1 and CBD-2. siRNA concentration = 2 µM; wavelength, 200–300 nm; bandwidth, 1.0 nm; response time, 4 s; scanning speed, 50 nm/min; step resolution, 0.5 nm. Na-phosphate buffer 10 mmol/L, pH 7.4. Results are presented as mean ± SD from a minimum of 3 independent experiments. * *p* < 0.05 compared to siRNA (after addition of CBD-1); # *p* < 0.05 compared to siRNA (after addition of CBD-2).

The ellipticity of complexes decreased with the higher dendrimer/siRNA molar ratio. The changes in θ/θ_0_ after addition of dendrimers to siRNAs are shown in Figure 3.

The changes in the siRNA CD spectra depended on the concentration of dendrimers. θ/θ_0_ decreased with an increasing concentration of CBD-1, finally reaching a stabilization. This indicates that the CBD-1 molecules gradually bind to both siRNAs until the saturation of the siRNA molecule at a 5:1 molar ratio (CBD-1:siRNA) occurs, whereas for CBD-2 there was decrease in the θ/θ_0_, but stabilization was not visible, probably because the dendrimer concentrations using this method were too low. Higher concentrations of dendrimers could not be used as they would interfere with the siRNA spectrum. Here, the system saturation was found for dendrimer CBD-1, but not for CBD-2, in contrast with the zeta potential, where CBD-1 needed more molecules to conjugate the siRNA.

### 2.3. Ethidium Bromide (EtBr) Intercalation Assay

**Figure 4:**
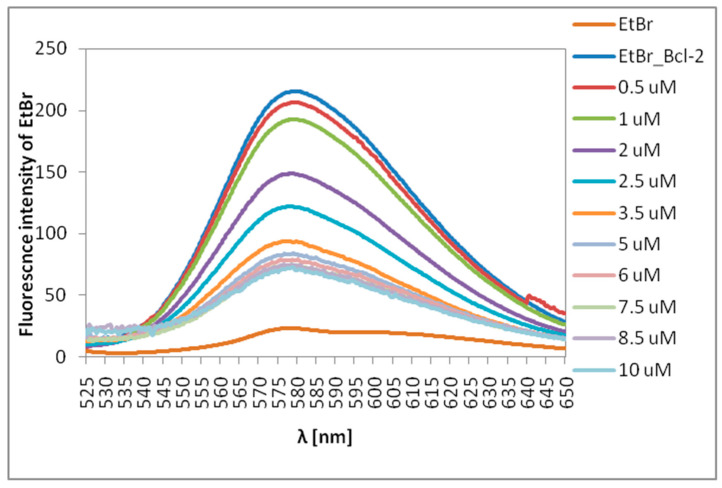
Dependence of the fluorescence intensity of the EtBr-Bcl-2 complex on an increasing concentration of CBD-2.

Fluorescence spectra of the EtBr-labeled Bcl-2 in the presence and absence of the CBD-2 dendrimer, as an example, are shown in Figure 4.

**Figure 5:**
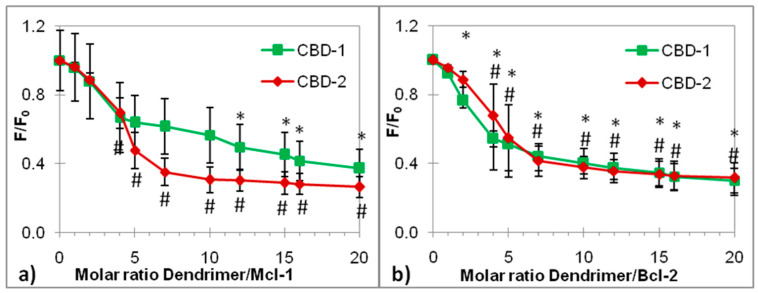
Dependence of the fluorescence intensity of the EtBr-siRNA complex on an increasing concentration of CBD-1 and CBD-2 dendrimers. (**a**) Mcl-1; (**b**) Bcl-2. siRNA concentration, 0.5 µM; EtBr concentration, 5 µM. Na-phosphate buffer, 10 mmol/L, pH 7.4. λex = 480 nm, λem = 525–650 nm. Results are presented as mean ± SD obtained from a minimum of 3 independent experiments. * *p* < 0.05 compared to siRNA (after addition of CBD-1); # *p* < 0.05 compared to siRNA (after addition of CBD-2).

Dendrimers without siRNA had no effect on the intensity of EtBr fluorescence, due to the lack of any EtBr/dendrimer interaction (data not shown). After the addition of dendrimers, the EtBr fluorescence decreased, which indicates the binding of the dendrimer to the siRNA and displacement of EtBr. Figure 5 shows changes of relative fluorescence intensity (F/F_0_) after the addition of dendrimers. The results proved the binding of CBD-1 and CBD-2 to Mcl-1 and Bcl-2. The relative fluorescence intensity (F/F_0_) was lower with the increasing dendrimer/siRNA molar ratio. For Bcl-2, the decline in F/F_0_ was similar, but differences in the shape of the curves were observed for Mcl-1.

### 2.4. Gel Electrophoresis

**Figure 6:**
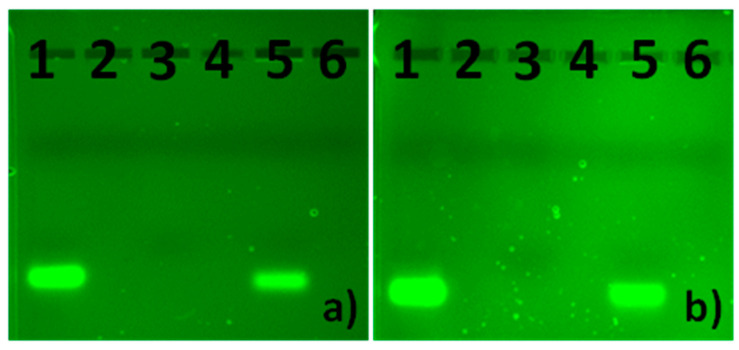
Protective effect of CBD-2 on siRNA in the presence of RNase A/T1 mix. (**a**) CBD-2 + Mcl-1, (**b**) CBD-2 + Bcl-2; 1, naked siRNA; 2, CBD-2; 3, siRNA + RNase; 4, siRNA + CBD-2 + RNase; 5, siRNA + CBD-2 + RNase + heparin; 6, siRNA + CBD-2.

Agarose gel electrophoresis helped to evaluate a possible protective effect of dendrimers on siRNA against degradation by RNase. Figure 6 shows photographs of gels after the electrophoresis of complexes of the CBD-2 dendrimer with Mcl-1 and Bcl-2. Naked siRNA was completely digested in the presence of RNase (Figure 6a,b, third lines). Treatment with RNase did not lead to degradation of siRNAs complexed with CBD-2 (Figure 6). Addition of heparin resulted in the release of siRNA from the dendriplexes and its migration through the gel (Figure 6a,b, fifth line).

### 2.5. XTT Assay

The MCF-7 cells were exposed to dendrimers and complexes of dendrimer/siRNA at different concentrations for 24 h. An XTT assay was also used for dimethyl sulfoxide (DMSO), the solvent. DMSO had no influence on the viability of the MCF-7 cells at dendrimer concentrations in the range of 0.1–5 µM, but it affected cells at the highest concentration, of 10 µM, decreasing viability to 60.5% vs. a negative control (NC).

**Figure 7:**
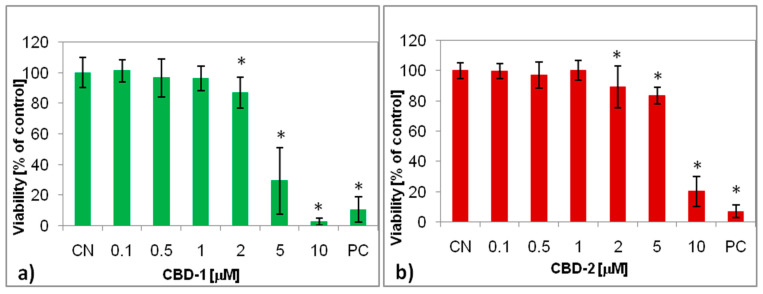

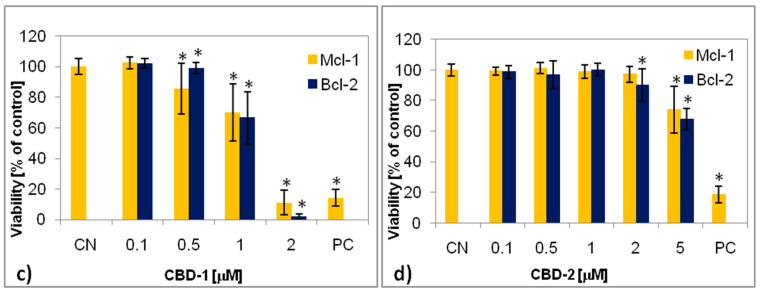
Viability of MCF-7 cells after 24 h exposure to dendrimers: CBD-1 (**a**) and CBD-2 (**b**) and dendriplexes (**c**,**d**) at different concentrations. The dendrimer/siRNA molar ratio was 20:1; * *p* < 0.05 compared to CN.

Both types of dendrimer decreased the cell viability in a dose-dependent manner (Figure 7). Compared to the control, a small, but statistically significant effect was observed at 2 µM for both the dendrimers and for CBD-2 at 5 µM. Significant cytotoxicity of CBD-1 was noted at 5 µM, with the viability reducing to 29.2%, whereas CBD-2 was cytotoxic at the higher concentration of 10 µM, reducing the viability to 20.0%. However, the toxicity of CBD-1 and CBD-2 might be related to the influence of the solvent at 10 µM.

The complexes of dendrimer/siRNA significantly decreased the cell viability in the case of CBD-1/siRNA. A cytotoxic effect was obtained at 1 µM, with viability reduced to 69.9 and 66.5% for complexes with Mcl-1 and Bcl-2, respectively. In contrast, the influence of the CBD-2/siRNAs complexes on viability, compared to the CBD-2 alone, was mild. The viability was reduced only at 5 µM to 73.9 and 67.7% for complexes with Mcl-1 and Bcl-2, respectively.

### 2.6. Cellular Uptake

**Figure 8:**
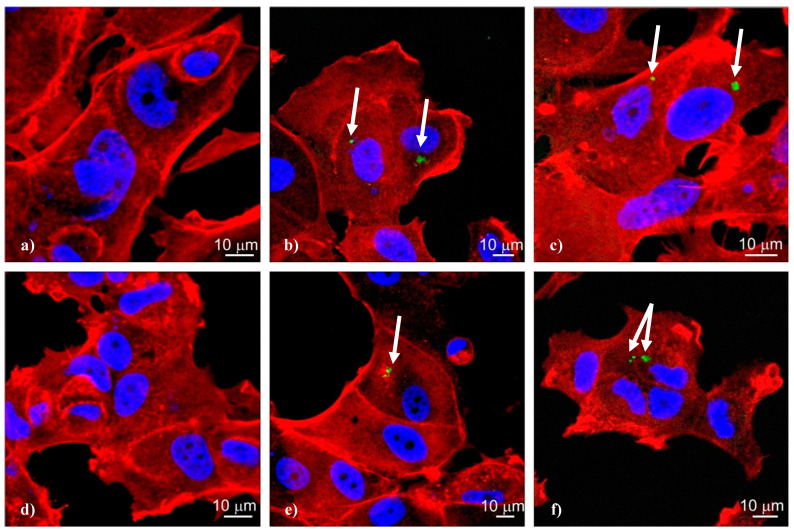
Confocal microscopy images of MCF-7 cells after 24-h incubation with fluorescein labelled siRNA: (**a**) Bcl-2, (**b**) CBD-1/Bcl-2, (**c**) CBD-2/Bcl-2, (**d**) Mcl-1, (**e**) CBD-1/Bcl-2, (**f**) CBD-2/Mcl-1. The concentration of siRNA was 0.1 µM and the dendrimer/siRNA charge ratio was 20:1. Arrows indicate complexes dendrimer/siRNA.

To analyze the ability of dendriplexes formed by CBD-1 and CBD-2 dendrimers with siRNA to become internalized in MCF-7 cancer cells after a 24-h incubation, confocal microscopy was used. Both the siRNAs (Mcl-1 and Bcl-2) complexed with CBD-1 and CBD-2 dendrimers entered the cells. The CBD/siRNA dendriplexes were visible in the cytoplasm as small green dots (Figure 8).

**Figure 9:**
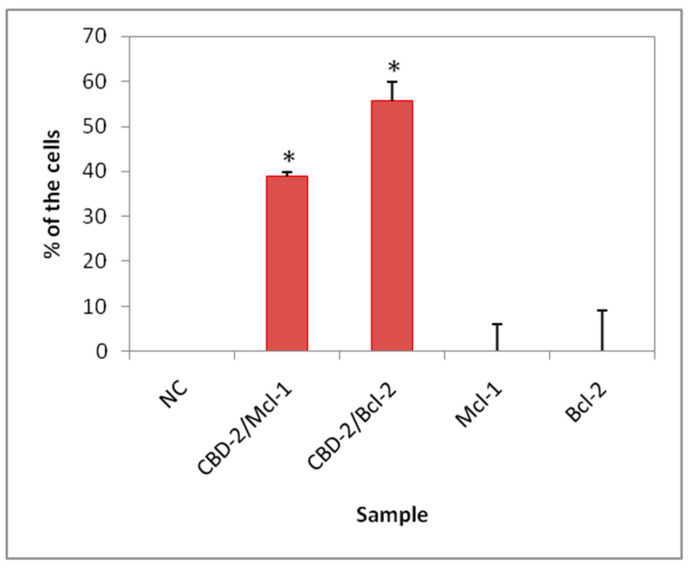
Percentage of cells containing dendrimer/siRNA complexes after 24-h exposure to dendriplexes or naked siRNA; NC, untreated cells. The dendrimer/siRNA charge ratio was 20:1, and the concentration of dendrimer was 2 µM; * *p* < 0.05.

An automatic microscope INCell Analyzer 2000 aided the quantification of the dendriplexes taken up by the cells. Analysis was carried out using CBD-2, and both examined siRNAs. The data show that 38.8 ± 5.9% of the cells (Figure 9) had CBD-2/Mcl-1 complexes in their cytoplasm, whereas 55.7 ± 9.0% of the cells show the presence of CBD-2/Bcl-2 complexes in the cytoplasm. After treatment with naked siRNAs, the fluorescence from the FITC-labeled siRNA was not found in the cells.

## 3. Discussion

RNAi is a promising tool in gene therapy. In this process, the target mRNA is neutralized, and gene expression is inhibited. The following two types of RNA molecules could be used to induce this process: microRNA (miRNA) and siRNA [20,21]. Since nucleic acids are very sensitive to enzymatic degradation, and because their transport through cell membranes is prevented due to being negatively charged, effective delivery systems are needed to overcome these problems [21,22,23,24]. Initially, viruses were considered as gene carriers in gene therapy because of their ability to transfect eukaryotic cells and silence gene expression. This system, however, has some disadvantages, which include the high cost of production, generation of certain side effects and induction of immune responses [21,25,26,27]. 

Nanomaterials, e.g., dendrimers, are promising as siRNA carriers in gene silencing during gene therapy [5,28,29,30]. For the first time, carbosilane dendrimers have been characterized as good carriers for siRNA by Weber et al. [31]. These dendrimers silenced glyceraldehyde 3-phosphate dehydrogenase (GAPDH) expression and reduced HIV replication in PBMC and human leukemia T lymphocytes, remaining at a low cytotoxicity level [16,31]. Posadas et al. [32] demonstrated that the carbosilane dendrimer 2G-NN16 can transport siRNA into neuronal cells and block the synthesis of the HIF1-*α* protein, and that the efficiency is similar to that achieved with viral vectors [32]. Krasheninina et al. [33] examined the complexation of anti-cancer siRNAs by second (BDEF32) and third (BDEF33) generation cationic carbosilane dendrimers and the influence of those complexes on two types of cancer cells, namely adherent HeLa cells and suspension HL-60 cells. Both types of dendrimers affected cell viability at 2.5, 5 and 10 µM (HeLa) or 5 and 10 µM (HL-60 cells). Moreover, dendrimers complexed with pro-apoptotic siRNAs increased cytotoxicity [33]. The methods used for the synthesis of dendrimers also offer wide possibilities in their design. Dendrimers can be modified as needed by changing their size, generation, charge density and the adding functional groups [31]. Therefore, in our experiments using carbosilane dendrimers CBD-1 and CBD-2 as potential anticancer siRNA (Mcl-1 and Bcl-2) carriers, we investigated the formation of complexes of dendrimer/siRNA, characterized them and evaluated their influence on MCF-7 viability and cellular uptake.

The formation of dendrimer/siRNA complexes was confirmed by the zeta potential measurement. This parameter changed significantly from negative to positive values with an increasing dendrimer/siRNA charge ratio. The positive charge of CBD-1 and CBD-2 dendrimers is responsible for penetration of cell membranes, allowing the internalization of the dendriplexes. The internalization of cationic dendrimers is more effective than anionic or neutral dendrimers [34]. It is also proven that positively charged dendrimers are more toxic than their analogs with a negative charge on the surface [35]. Cationic nanoparticles have the ability to damage the integrity of the cell membrane and can lead to the lysis of the cells by increasing their permeability [36]. Zeta potential measurements also helped to estimate the number of CBD molecules bound to one molecule of siRNA. The data show that both Mcl-1 and Bcl-2 can bind more molecules of CBD-1 (15 and 16, respectively) than CBD-2 (9 and 11, respectively). The partial deprotonation of tertiary ammonium groups (RNHMe2) is possible at the assayed pH, yielding a less cationic density on the surface of CBD-1. This could explain the higher number of molecules required to conjugate with siRNA.

The CD spectrum of the RNA duplexes of A-type is characterized by a positive peak at ~260 nm and a negative peak at ~210 nm [37], which were also seen in our data. The addition of dendrimers and the increase in the dendrimer/siRNA molar ratio reduced ellipticity, which confirmed the formation of complexes. This decrease could be due to a reduction in the absorbance of nucleosides in the dendriplexes [38]. The data show that dendrimers do not significantly affect the structure of siRNAs, because the characteristic A-form pattern is maintained. This corresponds to previous results describing dendrimer/siRNA interactions [39].

Interactions between dendrimers and siRNAs were also confirmed by using EtBr intercalation assay. The addition of dendrimers to siRNAs stained with EtBr strongly quenched its fluorescence intensity ratio F/F_0_, since the dendrimers compete with EtBr and displace it from siRNA. Our results correspond with the results of Ionov et al. [5], Michlewska et al. [38] and Krasheninina et al. [33], who showed the capability of carbosilane dendrimers to form dendriplexes with siRNA.

The gel electrophoresis of CBD-2 complexed with Mcl-1 and Bcl-2, in the presence of RNase A/T1 Mix and heparin, proved the ability of dendrimers to protect siRNA against nucleases. Naked siRNAs were digested, and no stripe was visible. CBD-2 protected siRNAs against degradation, and the addition of heparin allowed for the release and migration of siRNA (Figure 6). The properties of dendrimers to protect siRNA against RNase degradation were also shown in other studies for CBD [5,31].

The chemical and physical properties of dendrimers help them to interact effectively with organelles, biological membranes and proteins. Cationic dendrimers with positively charged groups have an ability to interact with negatively charged biological membranes, which leads to the internalization of the dendriplexes [5,36]. The analysis of confocal microphotographs showed that both types of dendrimers, CBD-1 and CBD-2, seem to be good carriers of siRNA. The images also show FITC-labeled CBD-1/siRNA and CBD-2/siRNA complexes inside cells. Quantitative analysis also shows that >50% of the cells could be transfected with CBD-2/Bcl-2 complexes.

In contrast to measuring cellular uptake, cytotoxicity assays demonstrated a difference between the tested dendrimers in relation to their ability to reduce the viability of cancer cells. CBD-1 appeared more potently cytotoxic than CBD-2. Both types of dendrimers contain eight surface cationic groups considered responsible for the cytotoxic effects of dendrimers [40]. However, the nature of each peripheral moiety is different. CDB-1 contains pH-dependent tertiary ammonium groups (RNHMe2Cl), whereas CBD-2 includes pH-stable quaternary ammonium groups (RNMe3I). Thus, the difference in cytotoxic activity could be ascribed to a different mechanism of action, e.g., the proton sponge effect for endosomal scape from RNHMe2Cl groups. Interestingly, after the incubation of the cells with dendrimer/siRNA complexes (CBD-1/Mcl-1 and CBD-1/Bcl-2), cytotoxicity was shifted toward the lower concentrations, i.e., 1 µM vs. 5 µM for CBD-1/siRNA and CBD-1 alone, respectively. The viability of the cells treated with complexes of siRNA with the second dendrimer tested, CBD-2, was at a similar level to the viability seen after treatment with the dendrimer alone. Our results are in contrast with some other studies showing that the dendriplex had less toxicity than the dendrimer alone [31,36]. One explanation is that a non-complexed dendrimer exposed all of its cationic groups. However, our results are consistent with the studies of Krasheninina et al. [33] that show the cytotoxicity of dendrimers complexed with pro-apoptotic siRNAs being higher than of dendrimer alone. In the context of anticancer therapy, it would be important to obtain the toxic potential after binding cationic dendrimers with siRNA.

Dendrimers are not the only nanoparticles that can transfer siRNA. Peña-González et al. [41] combined gold nanoparticles (AuNPs) with cationic carbosilane dendrons and examined their ability to transport siRNA against human immunodeficiency virus (HIV). Some advantages of AuNPs as carriers of genetic material are, e.g., biocompatibility, stability and low toxicity. The functionalization of AuNPs with cationic groups, as carbosilane dendrons, enables the compacting of nucleic acid and protects it from degradation. The data confirmed the dependence of this toxicity on the generation, which is characteristic for dendritic systems. Moreover, size determines the toxicity of dendrons [41].

Pędziwiatr-Werbicka et al. [21] used silver nanoparticles (AgNPs) modified with carbosilane dendrons to form complexes with Bcl-XL. The complexes were tested on lymphocytes to evaluate their effect on proliferation and on HeLa cells, and to examine cytotoxic potential. The modified AgNPs formed complexes with tested siRNAs, and protected it against degradation; furthermore, an efficient cellular uptake was observed. However, the binding of siRNA to nanoparticles did not increase their cytotoxicity [21].

## 4. Materials and Methods

### 4.1. Carbosilane Dendrimers

**Figure 10:**
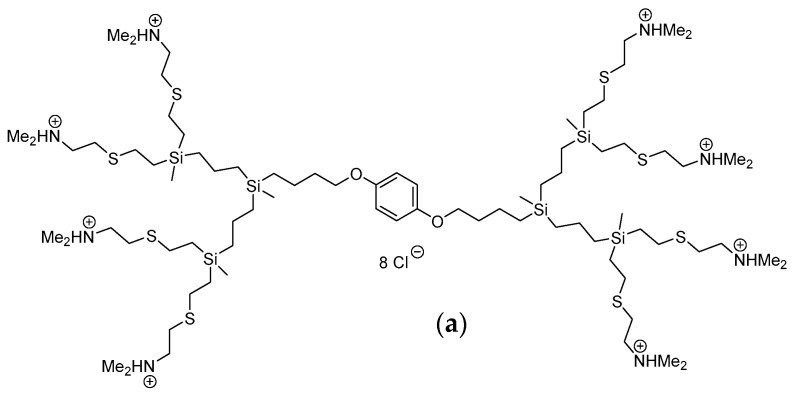

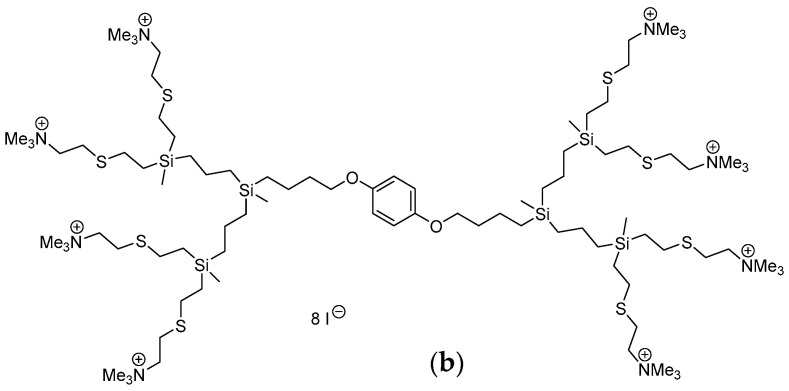
Molecular structure of carbosilane dendrimers: CBD-1 (**a**) and CBD-2 (**b**).

The new second generation carbosilane dendrimers, CBD-1 and CBD-2, were synthesized in the Department of Organic and Inorganic Chemistry, University of Alcalá, Madrid, Spain [42]. Carbosilane dendrimer CBD-1 contains 8 surface tertiary ammonium groups (RNHMe2Cl), Mw = 1997.01 g/mol; C80H182Cl8N8O2S8Si6. Carbosilane dendrimer CBD-2 contains 8 surface quaternary ammonium groups (RNMe3I), Mw = 2840.83 g/mol; C88H198I8N8O2S8Si6. Dendrimers are graphically presented in Figure 10. Dendrimers were dissolved in phosphate buffer (pH = 7.4) with 1% DMSO at 1 mM.

### 4.2. siRNA

Two types of anticancer siRNAs were used in this study: Mcl-1 and Bcl-2.
Mcl-1Sense Mcl-1s5′-GGACUUUUAUACCUGUUAUdTdT 3′Antisense Mcl-1a5′-AUAACAGGUAUAAAAGUCCdTdT 3′Bcl-2Sense E1s5′-G CUG CAC CUG ACG CCC UUCdTdT 3′Antisense E1a5′-GAA GGG CGU CAG GUG CAG CdTdT 3′

The siRNAs were purchased from Dharmacon Inc. (Lafayette, CO, USA).

### 4.3. Other Reagents

Reagents for cell culture were obtained from Biowest (Riverside, MO, USA. Dimethyl sulfoxide (DMSO), phosphate buffered saline (PBS) tablets, ethidium bromide, formaldehyde, ethylenediaminetetraacetic acid (EDTA) and heparin were obtained from Sigma Aldrich (St. Louis, MO, USA). 2,3-bis-(2-methoxy-4-nitro-5-sulfophenyl)-2H-tetrazolium-5-carboxanilide (XTT) was obtained from Biological Industries (Kibbutz Beit-Haemek, Israel). 4′,6-diaminido-2-phenylindole (DAPI), Texas Red-X Phalloidin and RNase A/T1 mix were purchased from Thermo Fisher Scientific (Waltham, MA, USA). Hoechst 33342 was obtained from Santa Cruz Biotechnology Inc. (Dallas, TX, USA). Tris was obtained from GE Healthcare (Chicago, IL, USA). Other basic chemical reagents (sodium dihydrogen phosphate, disodium hydrogen phosphate, acetic acid) were obtained from the local supplier, Chempur (Piekary Śląskie, Poland). Agarose was purchased from the local supplier, Blirt (Gdańsk, Poland). These chemicals were of analytical grade, and solutions were prepared using water purified using the Mili-Q system.

### 4.4. Dendrimer/siRNA Complexes Formation

Dendrimers and siRNAs were mixed in phosphate buffer (pH = 7.4) at concentrations giving the required molar ratio. Complexes were formed immediately.

### 4.5. Zeta Potential

The zeta potentials of the complexes were measured using a Malvern Instruments Zetasizer Nano-ZS (Malvern Instruments Ltd., Malvern, UK). Measurement was carried out at 25 °C. From 10 to 15 measurements were collected for each sample and averaged. Increasing concentrations of the dendrimer in the range of 0.5–20 µM were added to siRNA at 0.5 µM and the zeta potential was measured. The zeta potential value was calculated directly from the Helmholtz–Smoluchowski equation using Malvern software [43].

### 4.6. Circular Dichroism 

Structural changes of siRNAs (2 μM) in the presence of dendrimers at increasing concentrations (1–14 μM) were measured using the circular dichroism technique, using Jasco, J-815CD spectrometer (Tokyo, Japan) in 5-millimeter path length quartz cuvettes at 25 °C, with a wavelength step of 1 nm, a response time of 4 s and a scan rate of 50 nm/min. CD spectra were obtained between 300 and 200 nm. Each spectrum was reported as the average of 3 experiments. The dendrimer in phosphate buffer without siRNA was used as a baseline for dendrimer/siRNA complex spectra.

### 4.7. Ethidium Bromide (EtBr) Intercalation Assay

EtBr is a fluorescent dye that intercalates between nucleic base pairs. Dendrimers can compete with EtBr, bind to siRNA and lead to the displacement of EtBr, resulting in a decrease in its fluorescence [44,45,46].

Fluorescence of EtBr complexed with siRNA was measured using spectrofluorimetry with a Hitachi F-7000 at 25 °C (λ = 525–650 nm). EtBr was used at 5 µM and siRNA at 0.5 µM. Dendrimers were then added with increasing concentrations from 0.5–10 µM.

### 4.8. Gel Electrophoresis

To study the formation of complexes between dendrimers and siRNAs, and to investigate whether siRNA is protected from the degradation, agarose gel electrophoresis was used. Dendrimers (at 40 µM) were mixed with siRNA (final concentration 2 µM) in 10 mM Na-phosphate buffer, pH 7.4. RNase A/T1 mix (3 µg/mL) was added to evaluate the protective function of dendrimers. Samples were incubated for 30 min at 37 °C and then for 10 min on ice. After incubation on ice, heparin (0.082 mg/mL) was added. The samples were placed on agarose gel (3%) containing ethidium bromide. Electrophoresis was run in Tris-acetate-EDTA (TAE) buffer for 45 min at 90 V, 35 mA. The gel was visualized using ultraviolet (UV).

### 4.9. MCF-7 Cell Line

The MCF-7 cell line used in this study was purchased from American Type Culture Collection (ATCC^®^, cat. No.: HTB-22, Manassas, VA, USA). The cells were cultured in Dulbecco’s modified Eagle’s medium supplemented with 10% fetal bovine serum (FBS) and antibiotics. Cultures were maintained in an incubator with 5% CO_2_, at 37 °C.

### 4.10. XTT Assay

Cytotoxicity of dendrimers and dendriplexes was evaluated using the XTT assay, based on the ability of living cells to transform negatively charged tetrazolium salt into an orange color when reduced to a soluble formazan dye. Extracellular XTT reduction process is carried out by electron transport across the plasma membrane of living cells. The amount of living cells correlates with the coloration intensity measured spectrophotometrically. Test material is considered cytotoxic when viability is <70% vs. the negative control.

The MCF-7 cells were seeded onto 96-well plate at 5 × 10^3^ cells per well and incubated for 24 h before dendrimers (at 0.1, 0.5, 1, 2, 5 and 10 µM) or dendriplexes (dendrimer concentrations as above) were added. The molar dendrimer/siRNA ratio was 20:1. The cells grown in the supplemented culture medium were used as a negative control (NC). Triton X-100 at 0.01% was used as a positive control (PC). After 24 h, the XTT assay was performed according to the manufacturer’s procedure. Viability was calculated as a percentage of the NC.

### 4.11. Cellular Uptake

Complexes of dendrimers with FITC labelled siRNA were used to estimate the cellular uptake of dendriplexes. For confocal analysis, cells were seeded on microscopic slides placed at the bottom of 12-well culture plates. After 24 h, dendriplexes were added at the charge ratio dendrimer/siRNA 20:1, and the concentrations of dendrimers and siRNA were 2 and 0.1 µM, respectively. After a 24-h incubation with dendriplexes, the cells were fixed with 3.7% formaldehyde solution for 30 min and washed with PBS. DNA was stained with DAPI for 5 min (0.5 mg/mL) and Texas Red-X Phalloidin for F-actin staining (0.1 μg/mL). Images were taken with confocal laser scanning microscopy platform TCS SP8 (Leica Microsystems, Wetzlar, Germany) with an objective of 63×/1.40 (HC PL APO CS2, Leica Microsystems, Wetzlar, Germany) at the following excitation wavelengths: 405 nm (DAPI), 488 nm (FITC) and 565 nm (Texas Red-X Phalloidin).

For quantitative analysis of the uptake of dendriplexes, an automated microscope INCell Analyzer 2000 (GE Healthcare Life Science, Cardiff UK) was used. Cells were seeded onto 96-well plates at 3 × 10^3^ cells per well. Dendriplexes were added as described above. For these analyses, the CBD-2 dendrimer was chosen. Cells were then fixed with 3.7% formaldehyde solution and stained with Hoechst 33342 (1 µg/mL) and Texas Red-X Phalloidin (approximately 1.65 µM), and finally imaged with the INCell Analyzer 2000. The images were submitted to an automated analysis protocol. The number of cells containing dendrimer/FITC-labeled siRNA complexes was measured.

### 4.12. Statistical Analysis

The results are presented as mean ± SD. Data were analyzed by a one-way ANOVA test, followed by Tukey’s analysis, using Origin software. A value of *p* < 0.05 was accepted as statistically significant.

## 5. Conclusions

Measurements of the physical properties of dendriplexes (zeta potential, circular dichroism), electrophoresis and ethidium bromide assay have confirmed that both tested dendrimers complex with siRNA. Furthermore, confocal microscopy indicates that both dendrimers can effectively deliver siRNA into target cells. However, the XTT assay showed that cytotoxicity after incubation with tested dendriplexes was significantly higher for complexes with CBD-1. This suggests that, potentially, this dendrimer is more appropriate than CBD-2 as a siRNA carrier in gene therapy.
